# Effect of Synthesis and Processing Conditions on the Sintering Behavior and Total Conductivity of High-Entropy Fluorite/Bixbyite Oxides (RE-HEOs)

**DOI:** 10.3390/ma18112663

**Published:** 2025-06-05

**Authors:** Luca Spiridigliozzi, Viviana Monfreda, Antonello Marocco, Filippo Milano, Antonio Vendittelli, Gianfranco Dell’Agli

**Affiliations:** 1 Department of Civil and Mechanical Engineering, University of Cassino and Southern Lazio, Via G. Di Biasio 43, 03043 Cassino, Italy; viviana.monfreda@unicas.it (V.M.); antonello.marocco@unicas.it (A.M.); dellagli@unicas.it (G.D.); 2 National Interuniversity Consortium of Materials Science and Technology (INSTM), Via G. Giusti 9, 50121 Florence, Italy; 3 EUT + Institute of Nanomaterials and Nanotechnologies-EUTINN, European University of Technology, European Union, 03043 Cassino, Italy; 4 European University of Technology, European Union, 03043 Cassino, Italy; filippo.milano@unicas.it (F.M.); antonio.vendittelli@unicas.it (A.V.); 5 Department of Electrical and Information Engineering, University of Cassino and Southern Lazio, Via G. Di Biasio 43, 03043 Cassino, Italy

**Keywords:** high-entropy oxides, co-precipitation, hydrothermal treatment, sintering, rare-earth-based oxides

## Abstract

This study explores the influence of two different synthesis methods on the sintering behavior of three novel high-entropy oxides possibly suitable for thermal barrier applications: (Ce_0.2_Zr_0.2_Yb_0.2_Er_0.2_Nd_0.2_)O_2-δ_, (Ce_0.2_Zr_0.2_Yb_0.2_Er_0.2_La_0.2_)O_2-δ_, and (Ce_0.2_Nd_0.2_Yb_0.2_Er_0.2_La_0.2_)_2_O_3+δ_. Rare-Earth-based High-Entropy Oxides (RE-HEOs), recently known for their exceptional thermal stability and compositional flexibility, have gained increasing attention as potential candidates for many advanced technological applications. Thus, our current work focuses on the specific effects of synthesis techniques, namely co-precipitation and hydrothermal treatment, on the entropy-driven stabilization, microstructure, electrochemical properties, and sintering behavior of three novel RE-HEOs. The obtained results reveal significant differences in terms of densification yield and of the obtaining of the designed entropy-stabilized single phase depending on the adopted synthesis route, underscoring the critical role of synthesis in optimizing RE-HEOs for near-future technological applications.

## 1. Introduction

High-entropy oxides (HEOs) represent a novel class of advanced ceramic materials characterized by a single-phase crystalline structure mainly stabilized by configurational entropy, which is typically achieved by the incorporation of five or more cation species in near-equimolar concentrations [1,2,3]. Such entropy-driven stabilization occurring at high temperatures, i.e., when the enthalpy of formation (ΔH_f_) is positive and the entropy of formation (ΔS_f_) is positive and large enough to make the Gibbs free energy of formation (ΔG_f_ = ΔH_f_ − TΔS_f_) negative above a certain temperature [4], mostly let HEOs exhibit unique thermodynamic and structural properties that significantly differ from “conventional” solid solutions [5,6,7,8]. Among the various classes and related proposed technological applications of HEOs, the Rare-Earth-based HEOs (RE-HEOs) emerged for their exceptional thermal stability [9], reduced thermal conductivity [10,11,12], and superior chemical inertness [13,14], all major features for high-temperature applications [15,16,17,18].

Conventional high-temperature ceramic systems typically employ yttria-stabilized zirconia (YSZ) due to its technological properties, such as moderate thermal conductivity [19], favorable (compared to most metals) coefficient of thermal expansion (CTE) [20], and good fracture toughness [21]. Nevertheless, YSZ faces significant operational limitations at temperatures above approximately 1200 °C, where sintering-induced densification and polymorphism (i.e., tetragonal-to-monoclinic phase transformation) could significantly hinder coating performance [22,23,24]. Alternatively to YSZ, extensive research exploration [25,26,27,28] for high-temperature applications has been dedicated to rare-earth zirconates too, i.e., ternary oxides with the general formula RE_2_Zr_2_O_7_ exhibiting a pyrochlore structure. This class of compounds, in which RE represents either a single lanthanide element or a combination of multiple lanthanides, exhibits notably low thermal conductivity alongside remarkable thermal stability [29], making them suitable as promising materials capable of sustained performance at temperatures exceeding 1300 °C [30,31]. Furthermore, defect engineering proved to be highly effective in further reducing the intrinsic thermal conductivity of RE_2_Zr_2_O_7_ pyrochlores, potentially achieving values below approximately 1 W·m^−1^·K^−1^ [32].

However, RE-HEOs exhibiting fluorite-like structures or their derivatives (such as pyrochlore and bixbyite) have demonstrated even more promising characteristics for possible use in ultra-high-temperature applications such advanced thermal barrier coatings (TBCs) [33,34,35]. In fact, their inherently disordered crystal structure contributes to substantial phonon scattering, drastically reducing thermal conductivity and enhancing thermal insulation capabilities. Additionally, these disordered structures often exhibit significant defect tolerance and chemical flexibility, allowing fine-tuning of properties such as the thermal expansion coefficient or resistance to high-temperature degradation mechanisms, including corrosion from calcium-magnesium-alumino-silicate (CMAS) deposits [36].

The authors previously demonstrated that the adopted fabrication cycle of RE-HEOs critically influences their “formation paths” [37], as the reactivity of precursor RE-HEOs powders strongly affects their entropy-driven stabilization kinetics. Particularly, carbonate-based co-precipitation proved to be the best option (compared to different co-precipitation environments and to the conventional solid-state synthesis method) to obtain highly reactive powders that are entropy-stabilized at relatively low temperatures. However, hydrothermal treatment is also known for producing homogeneous precursor powders in rare-earth-based conventional systems [38,39,40] and even in differently-structured HEOs [41,42,43], consequently leading to very good densification levels and phase purity of the final sintered pellets.

Thus, as optimizing synthesis and sintering protocols is crucial for the possible near-future application of RE-HEOs, this study specifically investigates the impact of two distinct synthesis methods, i.e., co-precipitation and hydrothermal treatment, on the microstructure, entropy-stabilized phase formation, sintering behavior and (preliminarily) electrochemical features of three novel fluorite/bixbyite-structured high-entropy oxides designed using the standard deviation predictor proposed by Spiridigliozzi et al. [44]: (Ce_0.2_Zr_0.2_Yb_0.2_Er_0.2_Nd_0.2_)O_2-δ_, (Ce_0.2_Zr_0.2_Yb_0.2_Er_0.2_La_0.2_)O_2-δ_, and (Ce_0.2_Nd_0.2_Yb_0.2_Er_0.2_La_0.2_)_2_O_3+δ_, labelled as CZYbEN, CZYbEL, and CNYbEL, respectively.

To the best of our knowledge, this study provides the first systematic comparison between carbonate-based co-precipitation and hydrothermal treatment for the synthesis of RE-HEOs, demonstrating that the co-precipitation route not only enables full densification under relatively mild sintering conditions, but also yields systems with highly promising electrochemical performances in the intermediate temperature range.

## 2. Materials and Methods

Cerium (III) nitrate (Ce(NO_3_)_3_·6H_2_O, Carlo Erba Reagents S.r.l., Cornaredo, Lombardia, Italy), lanthanum (III) nitrate (La(NO_3_)_3_·6H_2_O, Merck Serono S.p.A., Rome, Lazio, Italy), ytterbium (III) nitrate (Yb(NO_3_)_3_·5H_2_O, Merck Serono S.p.A., Rome, Italy), neodymium (III) nitrate (Nd(NO_3_)_3_·5H_2_O, Merck Serono S.p.A., Rome, Italy), erbium (III) nitrate (Er(NO_3_)_3_·5H_2_O, Merck Serono S.p.A., Rome, Italy), and zirconium (IV) oxynitrate (ZrO(NO_3_)_2_, Carlo Erba Reagents S.r.l., Cornaredo, Italy) were used as precursors for the differently prepared samples, while ammonium carbonate ((NH_4_)_2_CO_3_, Merck Serono S.p.A., Rome, Italy) was used as a precipitating/mineralizing agent. All reagents were of a purity > 99% and were used without further purification.

Two distinct synthesis techniques were adopted within this work: co-precipitation and hydrothermal treatment. Regarding co-precipitation, the following procedure has been adopted for the synthesis of each HEO composition studied: (i) two aqueous solutions containing stoichiometric amounts of the selected nitrate salts and a proper amount of ammonium carbonate granting a molar ratio of anions to total rare-earth nitrates (R = [anions]/[total cations]) equal to 2.5, respectively, were kept under continuous stirring to ensure homogeneity; (ii) the solutions were mixed together to let co-precipitation occur (magnetic stirring was applied upon precipitation); (iii) the obtained precipitates were filtered via vacuum filtration and subsequently washed with deionized water; and (iv) the filtered precipitates were dried overnight at 60 °C.

The resulting dried co-precipitates powders were calcined at different temperatures (i.e., 800 °C, 1000 °C, and 1250 °C) for 1 h to induce entropy-driven stabilization of either the fluorite-like or bixbyite-like predicted structure. The co-precipitated samples were labelled CZYbEN-CP, CZYbEL-CP, and CNYbEL-CP.

In parallel, hydrothermal syntheses of the different HEFO systems were carried out as a possible alternative method to eventually achieve either more reactive precursors or already-crystalline systems. The same stoichiometric ratios of precursor nitrate salts as used in co-precipitation were dissolved in distilled water, thoroughly stirred, and transferred into Teflon vessels subsequently placed in sealed stainless steel outer vessels. The sealed vessels were subjected to hydrothermal treatment at a controlled temperature of 140 °C for 18 h, after which they were naturally cooled to room temperature.

The resultant precipitates were recovered via vacuum filtration, thoroughly washed with distilled water, and subsequently dried overnight at 60 °C. Similarly to the co-precipitated powders, the dried hydrothermally treated precursors were calcined at 800 °C, 1000 °C, and 1250 °C for 1 h to study entropy-driven stabilization of the expected fluorite-like or bixbyite-like structure. Hydrothermally treated samples were labelled as CZYbEN-HT, CZYbEL-HT, and CNYbEL-HT.

The thermal behaviors of both the CP and HT HEFO systems were analyzed by simultaneous differential thermal analysis and thermo-gravimetric analysis (DTA–TG) in air, using a-Al_2_O_3_ as a reference (Thermoanalyzer STA 409, Netzsch) and 10 °C/min as the heating rate.

Structural characterization was carried out on both as-synthesized and variously calcined HEFO systems through X-ray powder diffraction by using a Panalytical MPD X’PERT diffractometer (Cu Ka radiation).

Both CP and HT as-synthesized HEFO powders were subjected to direct sintering at two different temperatures, i.e., 1200 °C and 1300 °C, to assess their sintering behavior and analyze their microstructural features in view of possible technological applications of thermal barrier coatings.

The relative densities of the differently sintered samples were determined according to Archimedes’ principle using an analytical hydrostatic balance (Gibertini, sensitivity ± 0.0001 g).

The microstructure of the best sintered pellets was analyzed via scanning electron microscopy (SEM) using a Philips microscope (XL30).

Finally, electrochemical impedance spectroscopy (EIS) [45,46] measurements at open circuit voltage (OCV) were carried out on CZYbEL-CP-s1300 (being the best sintered system of the studied RE-HEOs) using a frequency response analyzer (Reference 3000, Gamry Instruments, Warminster, PA, USA) at a frequency range between 100 Hz and 1 MHz and an AC voltage amplitude of 100 mV. The EIS measurements were carried out in air within the 400–800 °C temperature range.

## 3. Results and Discussion

**Figure 1:**
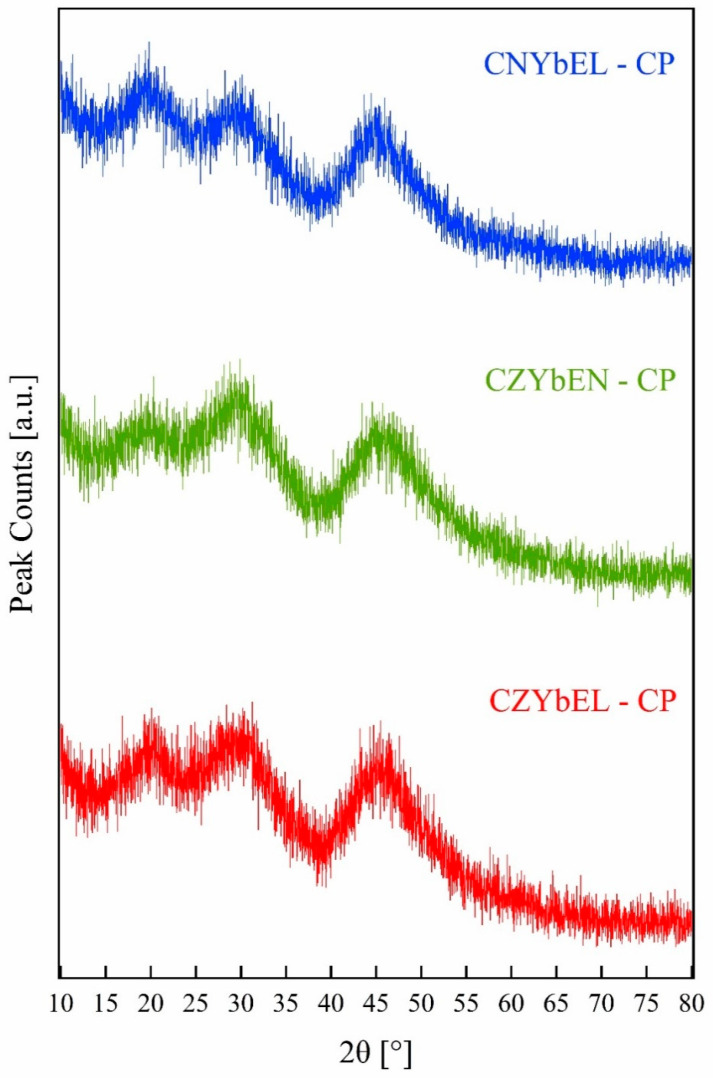
XRD patterns of the as-synthesized RE-HEOs-CP.

Figure 1 shows the XRD patterns of the co-precipitated RE-HEOs, i.e., CZYbEN-CP, CZYbEL-CP, and CNYbEL-CP. Irrespective of the actual composition, all the RE-HEOs-CP are fully amorphous, reflecting previously consolidated results regarding carbonate-based precipitation of rare-earth-based systems [47,48].

**Figure 2:**
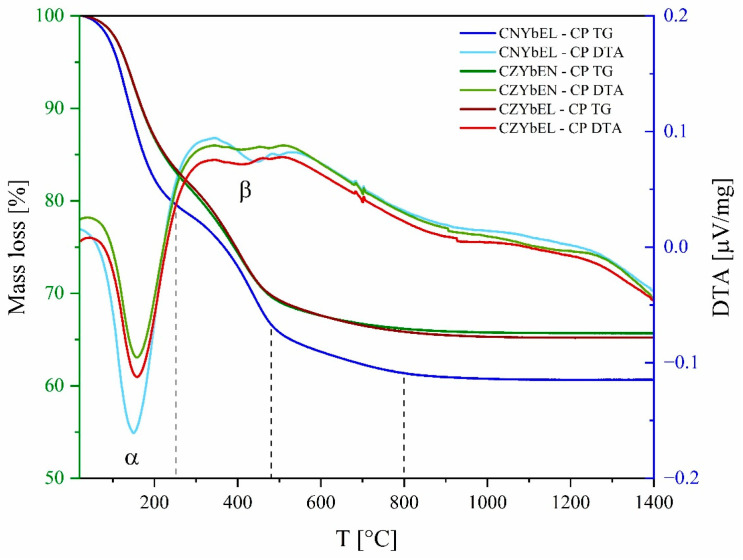
DTA-TG of the co-precipitated RE-HEOs. Irregularities in the DTA curves at about 700° and 900°C, respectively, are due to instrumental issues.

To assess the actual composition of the RE-HEOs-CP, simultaneous differential thermal analysis and thermo-gravimetric analysis (DTA–TG) were carried out on them. The resulting DTA-TG curves of the as-precipitated RE-HEOs are presented in Figure 2.

All three systems share a very similar thermal behavior, as they are characterized by two main thermal events (indicated as α and β on the DTA curves) upon heating. Particularly, the α endothermic event takes place at around 180 °C and represents water dehydration of the as-precipitated carbonates, coupled with a corresponding mass loss of roughly 15% for CZYbEN-CP and CZYbEL-CP and roughly 20% for CNYbEL-CP. The lower mass loss observed for the Zr-containing systems is likely due to its precipitation in the form of hydroxide rather than in the form of hydrated carbonates/hydroxycarbonates, as such mass loss of the former is significantly less than that of the latter.

Conversely, the β endothermic event takes place within a broader temperature range, i.e., around 300–500 °C, and can be associated with the amorphous carbonate/hydroxycarbonate decomposition, as already observed in similar systems [38]. Such thermal decomposition, occurring in two distinct macrosteps (the former in the 300–500 °C range and the latter, slower than the previous one, in the 500–800 °C range), is coupled with a corresponding mass loss of around 17% for all the three co-precipitated RE-HEOs. Above 800 °C, no additional thermal events are present in the three co-precipitated systems. Thus, based on previous results from multidoped ceria-based systems [48], we can suppose that the amorphous as-precipitated RE-HEOs are the following ones: $RE{CO}_{3}OH\cdot 2H_{2}O$ for CNYbEL-CP and a mixture of $RE{CO}_{3}OH\cdot 2H_{2}O$ and $Zr({OH)}_{4}$ for CZYbEN-CP and CZYbEL-CP (RE stands for a generic rare earth).

**Figure 3:**
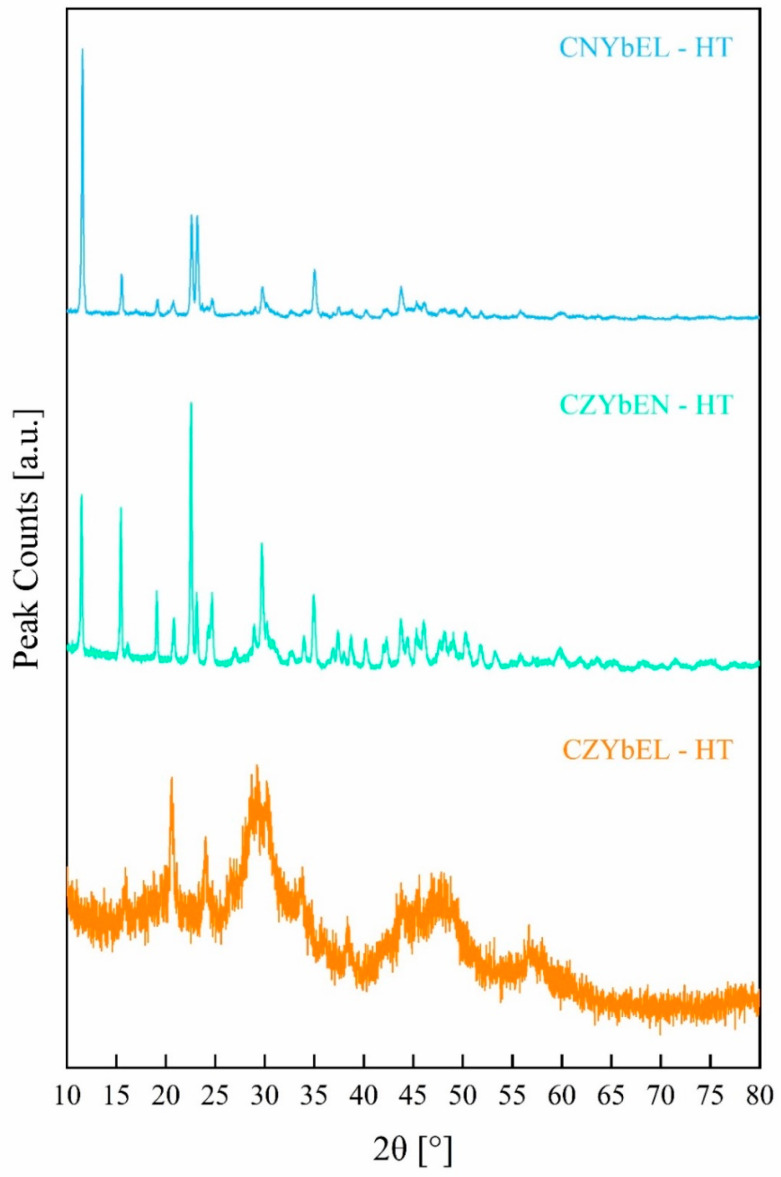
XRD patterns of the as-synthesized RE-HEOs-HT.

Figure 3 shows the XRD patterns of the hydrothermally synthesized RE-HEOs, i.e., CZYbEN-HT, CZYbEL-HT, and CNYbEL-HT.

Different from the as-precipitated systems, the three RE-HEOs-HT exhibit distinct structural features, being either fully crystallized (CNYbEL-HT and CZYbEN-HT) or partially crystallized (CZYbEL-HT).

Particularly, CNYbEL-HT and CZYbEN-HT are each formed by two different rare earth carbonates/hydroxycarbonates, namely a monoclinic RE(CO_3_)_3_·3H_2_O and an orthorhombic REOHCO_3_, where RE stands for a generic rare earth (ICDD card n. 00-052-1046 refers to Eu(CO_3_)_3_·3H_2_O, and ICDD card n. 01-070-2054 refers to NdOHCO_3_), very likely being solid solutions of the different rare earth cations, along with the presence of either amorphous or microcrystalline zirconium oxide barely detectable in Figure 3 in the form of small halos around 30° and 45–50°, typical of amorphous ZrO_2_ [49]. Conversely, CZYbEL-HT is a partially amorphous system (very likely formed by amorphous rare earth carbonates/hydroxycarbonates and zirconium hydroxide), presenting several broad peaks attributable to the bixbyite-like rare earth oxide RE_2_O_3_ (ICDD card n. 01-077-0458 referred to Yb_2_O_3_) too. To be more precise, even the CZYbEN-HT diffraction pattern exhibits a very small amorphous halo (not detected in the Zr-free CNYbEL-HT system) in the 29–33° range, very likely indicating the presence of a small portion of amorphous zirconium hydroxide.

**Figure 4:**
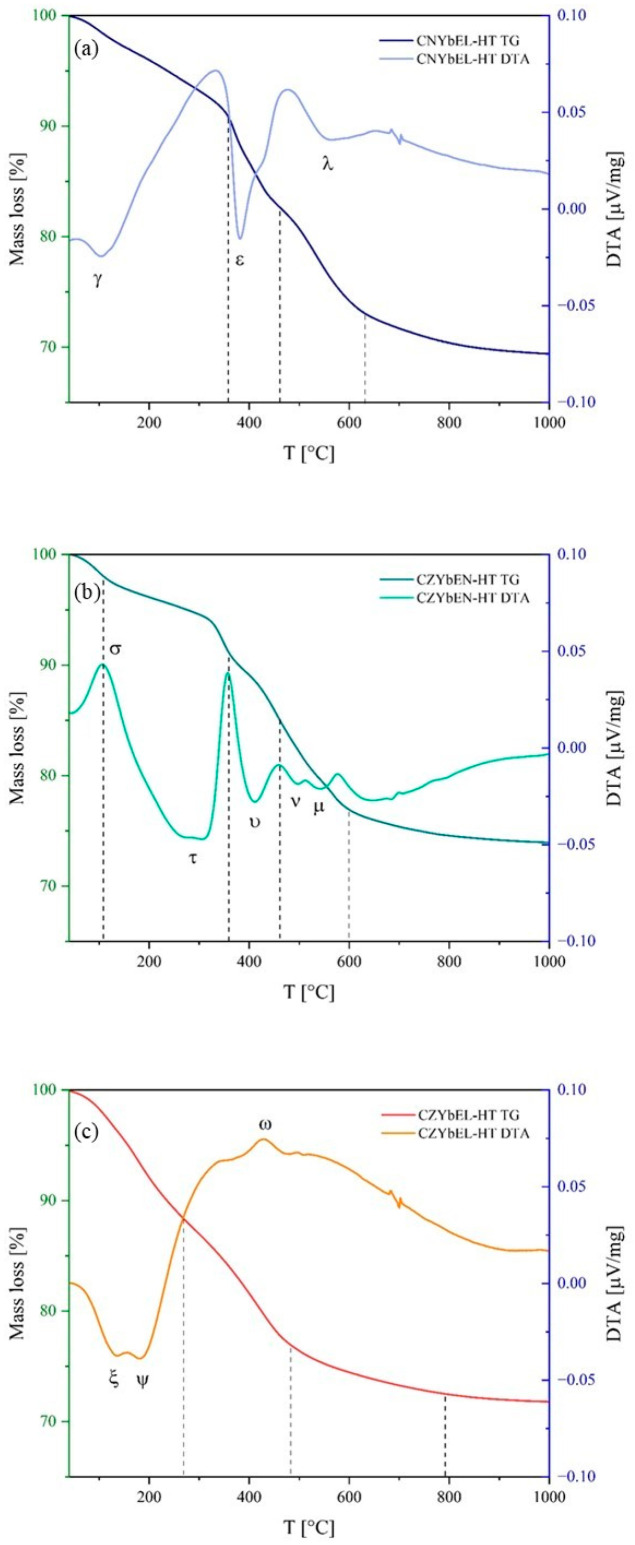
DTA-TG of the hydrothermally synthesized RE-HEOs: CNYbEL-HT (**a**), CZYbEN-HT (**b**), and CZYbEL-HT (**c**). Irregularities in the DTA curves at about 700° are due to instrumental issues.

According to their structural features, the thermal behaviors of the as-synthesized RE-HEOs-HT are much more complex than the corresponding RE-HEOs-CP systems. Figure 4 shows the DTA-TG curves of the hydrothermally synthesized RE-HEOs.

Here, each RE-HEO-HT system exhibits a different thermal behavior, reflecting their complex multiphasic natures. Particularly, CNYbEL-HT exhibits three distinct endothermic thermal events (indicated as γ, ε, and *λ* in Figure 4a) and three corresponding “decomposition intervals” up to around 650 °C. The first γ event is related to rather slow water evolution in the differently-hydrated carbonates/hydroxycarbonates up to around 350 °C; the second one (i.e., the ε event) occurs in the 350–450 °C temperature range, representing the first decomposition step of a generic $RE{CO}_{3}OH\cdot xH_{2}O$; and the last one (i.e., the λ event) represents the second decomposition step of a generic $RE{CO}_{3}OH\cdot xH_{2}O$ [38]. CZYbEN-HT, being formed by relatively even quantitative distribution of the carbonate-based phases, exhibits the most complex thermal behavior among the RE-HEOs-HT, as it is possible to distinguish (apart from the s event associated with carbonates/hydroxycarbonates dehydration) four endothermic thermal events (labelled with τ, υ, ν, and μ in Figure 4b) attributable to single-cation carbonate-based species, among which the two-step decomposition of a generic $RE{CO}_{3}OH\cdot xH_{2}O$ is still identifiable. Finally, CZYbEL-HT shows thermal behavior resembling RE-HEOs-CP systems, but with two main differences: (i) the rare earth carbonate/hydroxycarbonate dehydration occurring in the 100–300 °C temperature range is split in two separate steps, one at around 170 °C (ζ event) and the other above 170 °C (Ψ event); and (ii) the typical endothermic peaks associated with rare earth carbonate/hydroxycarbonate decomposition is “hindered” by an exothermic thermal event (ω event) accounting for the ZrO_2_ crystallization occurring at around 430 °C [49,50]. For the hydrothermally synthesized RE-HEOs, no additional thermal events above 800 °C have been observed, indicating full completion of the precursors’ thermal decomposition.

**Figure 5:**
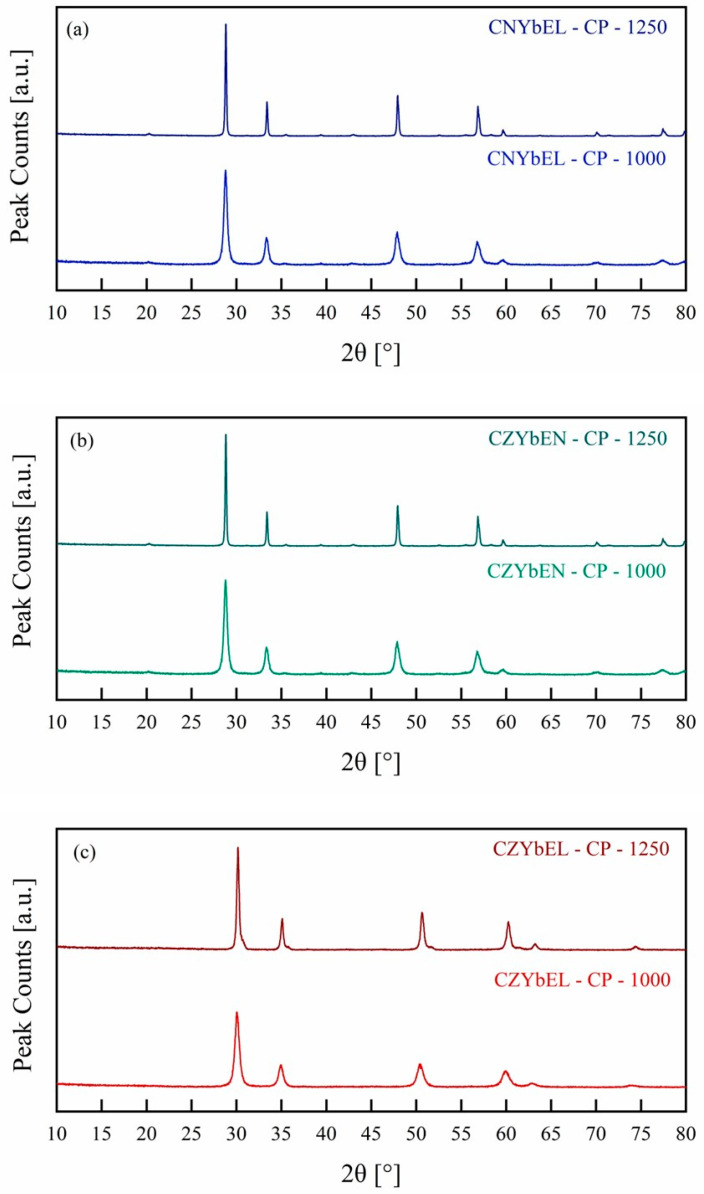
XRD patterns of the differently calcined RE-HEOs-CP: CNYbEL-CP (**a**), CZYbEN-CP (**b**), and CZYbEL-CP (**c**).

**Figure 6:**
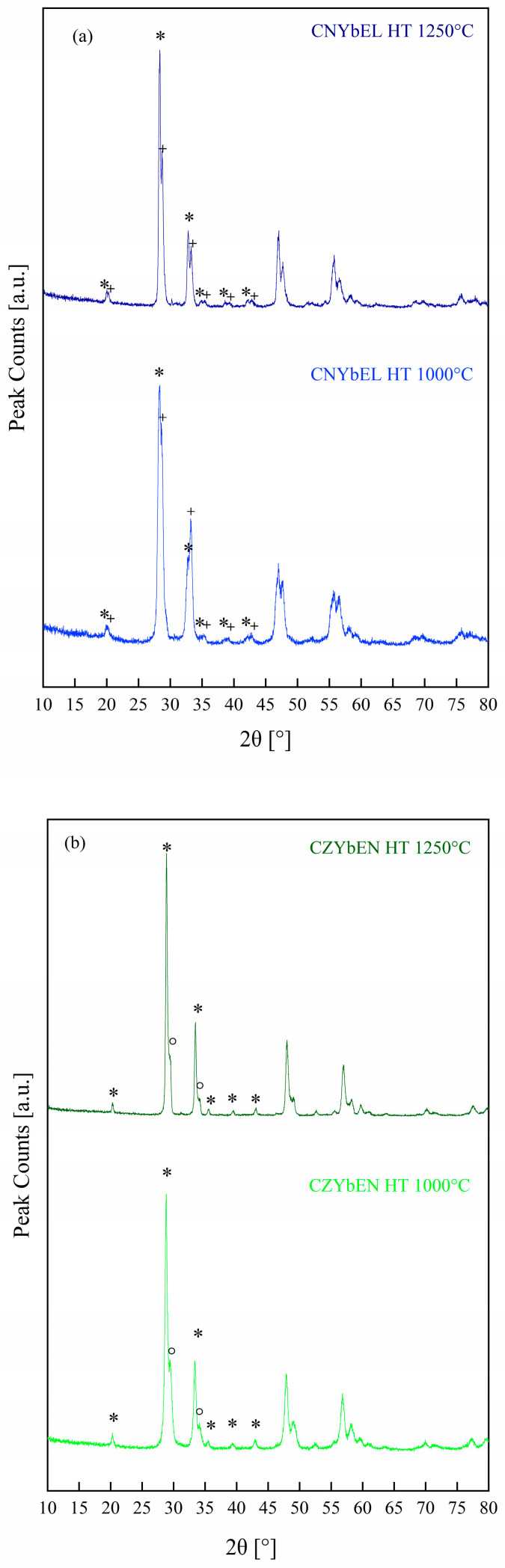

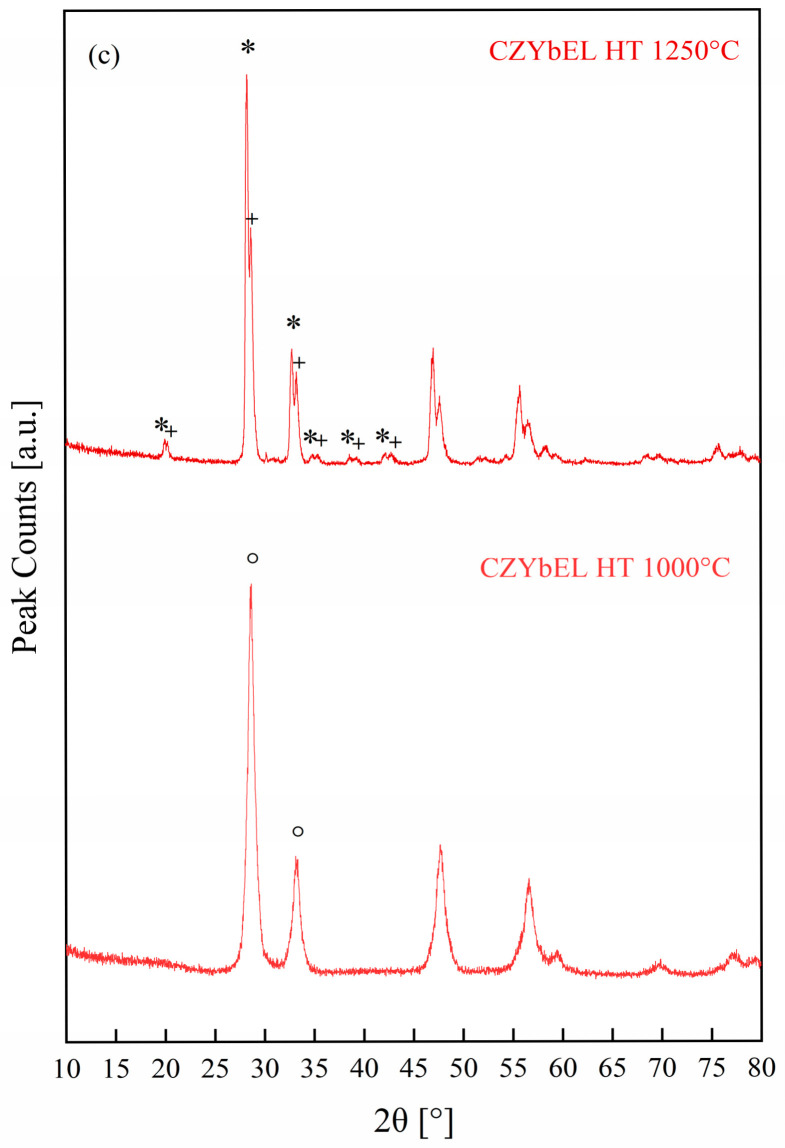
XRD patterns of the differently calcined RE-HEOs-HT: CNYbEL-HT (**a**), CZYbEN-HT (**b**), and CZYbEL-HT (**c**). * and + denote the main peaks of two distinct bixbyite-like phases, while ° denote the main peaks of a fluorite-like phase.

Based on the DTA-TG results, two different calcination temperatures were chosen to assess entropy-driven stabilization of the desired bixbyite/fluorite-like single phase in both co-precipitated and hydrothermally synthesized systems, i.e., 1000 °C and 1250 °C. In all cases, a heating rate of 10 °C/min and a soaking time of 1 h were used. Figure 5 and Figure 6 show the diffraction patterns of differently calcined RE-HEOs-CP and RE-HEOs-HT, respectively.

Clearly, two different behaviors are observable in the RE-HEOs-CP and RE-HEOs-HT, as, for the co-precipitated systems, either the predicted, according to the standard deviation predictor for RE-HEOs [43], bixbyite-like (CNYbEL and CZYbEN) or fluorite-like (CZYbEL) structure has been obtained at both calcining temperatures, with only visible crystallite growth occurring upon increasing the calcination temperature. The fluorite-like reference structure refers to a pure cerium oxide (ICDD card n. 01-089-8436), while the bixbyite-like reference structure refers to a pure terbium oxide (ICDD card n. 01-086-2478). The crystallite size for calcined RE-HEOs-CP has been calculated according to the Scherrer equation:

$$d=\frac{K\lambda }{B\cos\left(\theta \right)}$$

where *K* is the shape factor (0.89 for spherical particles), *λ* is the X-ray wavelength (0.1541 nm for Cu K*α*1), *θ* is the peak Bragg’s angle, and B is the relative full width at half maximum (FWHM) corrected for the instrumental broadening (determined with a standard polycrystalline silicon sample). Table 1 shows the resulting crystallite size for all the calcined RE-HEOs-CP.

Conversely, all the calcined RE-HEOs-HT are biphasic systems, either consisting of two distinct bixbyite-like phases (whose main peaks are labelled with * and + in Figure 6) or a bixbyite-like and a fluorite-like phase (whose main peaks are labelled with ° in Figure 6). Interestingly, CZYbEL-HT (see Figure 6c) exhibits destabilization of a metastable entropy-stabilized fluorite-like phase formed in CZYbEL-HT-1000 at higher calcination temperatures (i.e., 1250 °C).

Definitely, although hydrothermal synthesis is widely recognized for yielding highly reactive and pure powders in conventional, compositionally simpler systems, its effectiveness diminishes in the presence of high compositional complexity. Particularly for our RE-HEOs systems, the mild hydrothermal conditions selected were insufficient for obtaining the designed entropy-stabilized single phase upon mild calcination cycles (i.e., < than 1300 °C) and, consequently, they have not been subjected to any additional characterization. Finally, harsher hydrothermal conditions were deliberately avoided, given that co-precipitation already proved more effective for obtaining entropy-stabilized and easily sinterable RE-HEOs.

Once again, carbonate-based precipitation has proven to be the most effective synthesis method to produce RE-HEOs, as hydrothermal treatment, often a better option for obtaining high-quality precursors in “conventional” (i.e., non-high-entropy) rare-earth-based systems [38], led to a significantly slower “formation path”, similarly to ammonia-based precipitation and solid-state synthesis [37]. Thus, based on structural analysis of both RE-HEOs-CP and RE-HEOs-HT, a dedicated sintering protocol has been developed to maximize the densification of RE-HEOs-CP, firstly testing two different sintering temperatures, i.e., 1200 °C and 1300 °C (chosen for being close to the calcination temperature of 1250 °C, leading to well-crystallized single-phase RE-HEOs-CP), starting from the as-precipitated powders and, subsequently, measuring the pellets’ densities via Archimedes’ principle and comparing the obtained values with the crystallographic (theoretical) density of the various RE-HEOs. For both sintering cycles, a constant heating rate of 10 °C/min and a soaking time of 3 h have been used. Starting from the calculated parameters [51] for CNYbEL-CP-1250, CZYbEN-CP-1250, and CZYbEL-CP-1250 of 10.8811 Å, 10.7315 Å, and 5.3797 Å, respectively, their theoretical density ρ has been calculated according to the following crystallographic equation:

$$\rho =\frac{Z\sum_{i}\nu_{i}\cdot M_{i}}{a^{3}\cdot N_{A}}$$

where Z is the number of formula units per unit cell (4 for fluorite-like structure and 16 for bixbyite-like structure), ν*_i_* is the stoichiometric coefficient of the *i*th element, *M_i_* is the molar mass in g·mol^−1^ of the *i*th element, *a* is the lattice parameter, and *N_A_* is the Avogadro constant.

Table 2 summarizes the results obtained in terms of theoretical densities, measured densities, and relative densities of all three differently sintered RE-HEOs-CP (along with the calculated lattice parameters of CNYbEL-CP-1250, CZYbEN-CP-1250, and CZYbEL-CP-1250).

Two interesting results can be derived from analysis of Table 2: (i) RE-HEOs containing Zr required 1300 °C to complete their sintering process, as CZYbEN-CP-s1200 and CZYbEL-CPs1200 achieved relative densities as high as 70%, indicating a sintering process probably at the intermediate (second) stage [52] yet to be completed; and (ii) all the RE-HEOs-CP are practically fully dense (the “above 100%” value obtained for CZYbEN-CP-s1300 could be derived by a slightly different chemical composition compared to the nominal equimolar value, as assessed by CZYbEN-CP-s1300 EDS characterization, the results of which are reported in the Supplementary Materials) with a relatively mild sintering cycle (if compared with similar fluorite-structured-non high-entropy systems [53,54]).

**Figure 7:**
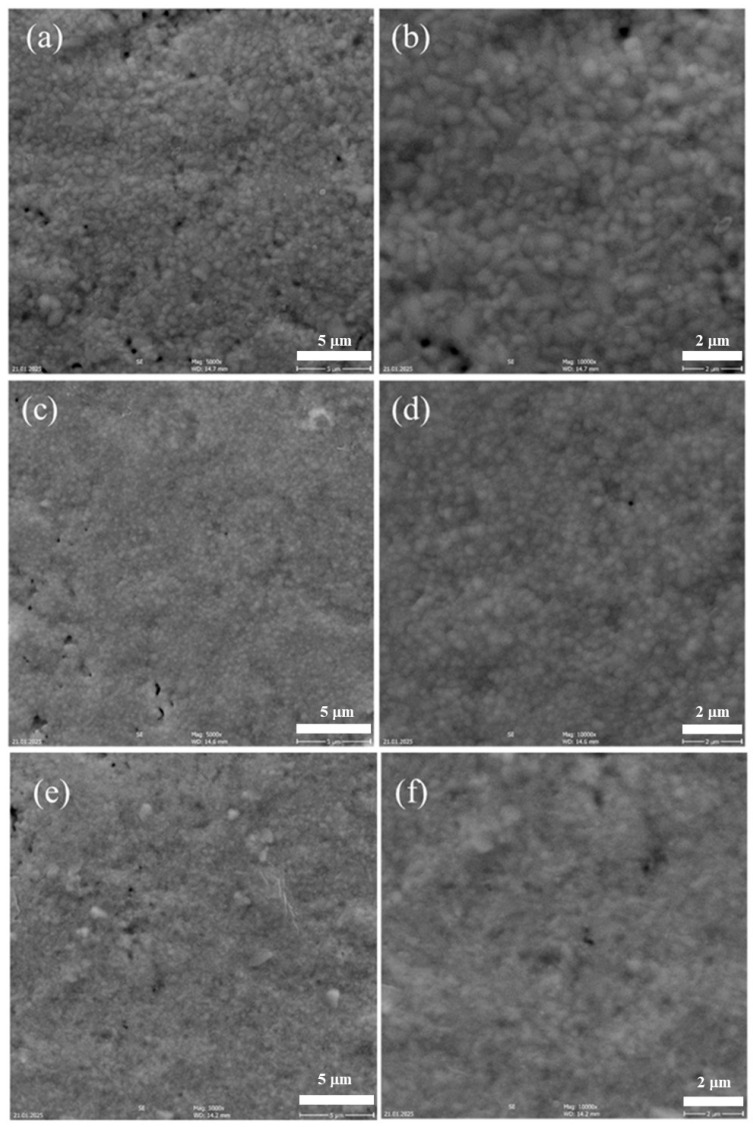
SEM micrographs of the sintered RE-HEOs-CP at different magnifications: CNYbEL-CP-s1300 (**a**,**b**), CZYbEN-CP-s1300 (**c**,**d**), and CZYbEL-CP-s1300 (**e**,**f**).

Figure 7 shows some representative micrographs, taken at different magnifications, of the RE-HEOs-CP sintered at 1300 °C for 3 h, revealing very dense microstructures with very few homogeneously distributed pores in all cases. Notably, Figure 7b, referred to CZYbEN-CP-s1300, shows a sample area of around 150 mm^2^ with just one visible pore. Thus, the density measurements reported in Table 2 are in perfect agreement with the SEM micrographs. Finally, both the average grain size and the residual porosity of the RE-HEOs-CP sintered at 1300 °C, estimated using the average grain intercept method according to [55] and ImageJ software, version 1.54k, are reported in Table 3. The residual porosity values estimated using ImageJ software well align with the measured densities reported in Table 2.

**Figure 8:**
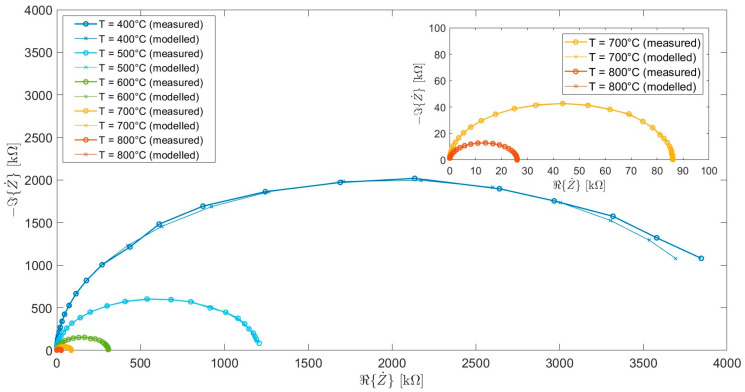
Measured and modelled Nyquist plots of CZYbEN-CP-s1300 at different temperatures (i.e., in the range 400–800 °C).

Finally, the electrochemical properties of CZYbEN-CP-s1300 were investigated through EIS, mainly to evaluate its total conductivity at temperatures ranging from 400 °C to 800 °C, i.e., a typical temperature range for ceria-based possible electrolytes for electrochemical devices [56]. The impedance responses, presented as Nyquist plots at such different temperatures, are reported in Figure 8 and its inset. Furthermore, the experimental data were fitted using an equivalent electrical model based on the well-known Randles circuit, consisting of series resistance of the electrolytic solution $\left(R_{s}\right)$ connected to a parallel resistor $\left(R_{p}\right)$ and a capacitor $\left(C_{p}\right),$ which model the electrode/electrolyte interface. Figure 8 also shows the impedance spectra modeled by the equivalent electrical circuit. The fitting operation consists in minimizing an objective function $F\left(\vartheta \right)$ that represents the difference between the modeled impedances ${\dot{Z}}_{model}$ and the measured ones ${\dot{Z}}_{meas}$ at the investigated frequencies. The modeled impedances are a function of the variable ϑ that groups the unknowns of the minimization problem, i.e., the electrical parameters of the Randles circuit: $\vartheta =\left\{R_{s},R_{p},C_{p}\right\}$. The adopted definition of the objective function is as follows:

$$F\left(\vartheta \right)=\sum_{i=1}^{N}\sqrt{{R\left\{{\dot{Z}}_{model,\;i}\left(\vartheta \right)-{\dot{Z}}_{meas,\;i}\right\}}^{2}+{I\left\{{\dot{Z}}_{model,\;i}\left(\vartheta \right)-{\dot{Z}}_{meas,\;i}\right\}}^{2}\;}$$

where $R\left\{\cdot \right\}$ and $I\left\{\cdot \right\}$ denote, respectively, the real and imaginary part of the difference between the two impedances and N represents the number of investigated frequencies. Finally, the parameter values are obtained by solving the problem $\hat{\vartheta }=\arg\underset{\vartheta }{\min}F\left(\vartheta \right)$ in the MATLAB environment using the simplex method [57], where $\hat{\vartheta }$ denotes the values of the obtained electrical parameters reported in Table 4 for the considered temperatures.

Across the studied temperature range (400–800 °C), the Nyquist plots only display a prominent semicircle associated with the electrode/electrolyte interface processes and a pure high-frequency resistance attributable to the overall CZYbEN-CP-s1300 response. Due to experimental limitations in isolating ionic from electronic conductivity at elevated temperatures (possibly occurring in CZYbEN-CP-s1300 due to the presence of Ce), only the total conductivity was considered in our preliminary electrochemical characterization. In fact, as previously and widely reported for ceria-based systems, the presence of Ce easily leads to a partial reduction under high-temperature conditions (Ce^4+^ → Ce^3+^), resulting in a non-negligible electronic contribution to total conductivity, especially in oxidizing atmospheres, thus complicating the accurate separation of ionic and electronic components in impedance-based measurements involving Ce-based systems [58,59,60,61].

**Figure 9:**
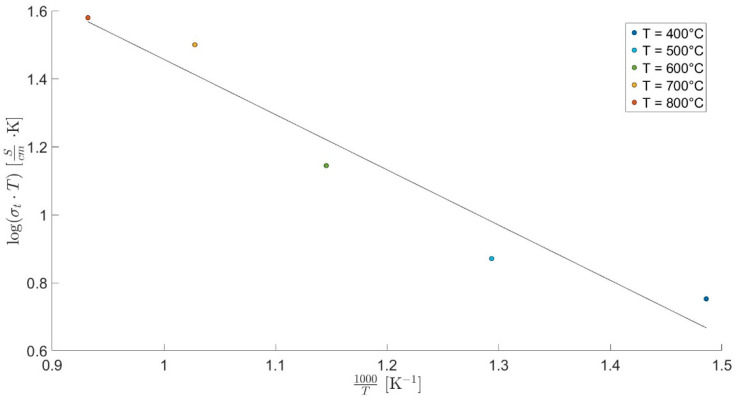
Nyquist plots of CZYbEN-CP-s1300 at different temperatures (i.e., in the range 400–800 °C).

An Arrhenius plot illustrating the linear relationship between log(σT) and 1/T is presented in Figure 9. 

The total conductivity observed for the CZYbEN-CP-s1300 sample at different temperatures, reported in Table 5, is well-aligned with conductivities found in the literature for highly performing ceria-based systems [55], suggesting that rare-earth-based high entropy oxides are potentially suitable as solid electrolytes for low/intermediate-temperature electrochemical devices.

## 4. Conclusions

The present study has investigated the influence of two different synthesis methods, i.e., carbonate-based co-precipitation and hydrothermal treatment, on the formation and sintering behavior of three novel rare-earth-based high-entropy oxides (RE-HEOs), either fluorite- or bixbyite-structured. The selected compositions, specifically (Ce_0.2_Zr_0.2_Yb_0.2_Er_0.2_Nd_0.2_)O_2-δ_, (Ce_0.2_Zr_0.2_Yb_0.2_Er_0.2_La_0.2_)O_2-δ_, and (Ce_0.2_Nd_0.2_Yb_0.2_Er_0.2_La_0.2_)_2_O_3+δ_, were rationally designed through the application of a configurational-entropy-based structural predictor previously proposed by the authors and subsequently synthesized and processed under similar conditions.

The obtained results revealed that co-precipitation leads to fully amorphous precursors, favoring the rapid formation of homogeneous, entropy-stabilized single-phase structures at relatively low calcination temperatures (i.e., starting from 1000 °C). In contrast, hydrothermal synthesis yielded multiphasic systems with slower transformation kinetics, despite it usually producing very reactive powders in conventional ceramics, especially in rare-earth-based systems. Consequently, co-precipitation proved to be a more suitable route for achieving structural homogeneity and phase purity in RE-HEOs.

Among all investigated systems, co-precipitated RE-HEOs, particularly CZYbEN-CP and CZYbEL-CP, achieved near-full densification at 1300 °C, with excellent microstructures formed of submicrometric grains and uniformly distributed in size.

Finally, preliminary electrochemical impedance spectroscopy (EIS) measurements performed on CZYbEN-CP-s1300 revealed highly promising total ionic conductivity in the 400–800 °C range, which is compatible with application in intermediate-temperature electrochemical devices. Although further work is needed to deconvolute ionic and electronic contributions, the current data already suggest significant functional behavior for future technological applications of RE-HEOs.

Overall, our work highlights the crucial role of synthesis route in governing both the entropy-driven stabilization and the final sintering behavior of RE-HEOs, thereby suggesting an optimized fabrication protocol for multifarious RE-HEOs.

## Figures and Tables

**Table 1:** Crystallite size of the variously calcined RE-HEOs-CP.

Calcination Temperature	CNYbEL-CP	CZYbEN-CP	CZYbEL-CP
**1000 °C**	33 nm	24 nm	19 nm
**1250 °C**	60 nm	102 nm	63 nm

**Table 2:** Theoretical, measured, and relative densities of the variously sintered RE-HEOs-CP.

RE-HEO System	Theoretical Density [g/cm^3^]	Measured Density [g/cm^3^]	Relative Density
**CNYbEL-CP-s1200**	7.3548	7.1859	97.7%
**CNYbEL-CP-s1300**	7.3548	7.2001	97.9%
**CZYbEN-CP-s1200**	7.3254	5.1552	70.4%
**CZYbEN-CP-s1300**	7.3254	7.3449	≈100%
**CZYbEL-CP-s1200**	7.2231	4.8851	67.6%
**CZYbEL-CP-s1300**	7.2231	7.0406	97.5%

**Table 3:** Theoretical, measured and relative densities of the variously sintered RE-HEOs-CP.

RE-HEO System	Average Grain Size [mm]	Residual Porosity [%]
**CNYbEL-CP-s1300**	0.50	5.0%
**CZYbEN-CP-s1300**	0.30	1.5%
**CZYbEL-CP-s1300**	0.25	3.5%

**Table 4:** Data fitting for Nyquist plots of CZYbEN-CP-s1300 reported in Figure 8.

Circuit Parameters	Temperature [°C]				
	400	500	600	700	800
$R_{s}[Ω]$	8.93	7.81	4.69	2.31	2.12
$R_{p}[MΩ]$	4.01	1.20	0.31	0.08	0.02
$C_{p}[pF]$	115.67	114.96	118.97	114.34	114.11

**Table 5:** Data fitting for Nyquist plots of CZYbEN-CP-s1300 reported in Figure 8.

	Temperature [°C]				
	400	500	600	700	800
**Total conductivity [S/cm]**	8.4 × 10^−3^	9.6 × 10^−3^	1.6 × 10^−2^	3.2 × 10^−2^	3.5 × 10^−2^

## Data Availability

Data will be available on request.

## Supplementary Materials

The following supporting information can be downloaded at: https://www.mdpi.com/article/10.3390/ma18112663/s1, Figure S1: EDS maps of the exemplary CZYbEN-CP-s1300.
