# Supplementary material for: Effect of Synthesis and Processing Conditions on the Sintering Behavior and Total Conductivity of High-Entropy Fluorite/Bixbyite Oxides (RE-HEOs)
Source: Materials (Basel). 2025 Jun 5;18(11):2663. doi: 10.3390/ma18112663 (PMC12156929; doi:10.3390/ma18112663)
Supplement: Supplementary file 1 [file materials-18-02663-s001.zip › Supplementary Materials-materials-3661008.pdf]

On the effect of the synthesis and processing conditions on sintering behavior and total conductivity of High-Entropy Fluorite/Bixbyite Oxides (RE-HEOs) – Supplementary Materials

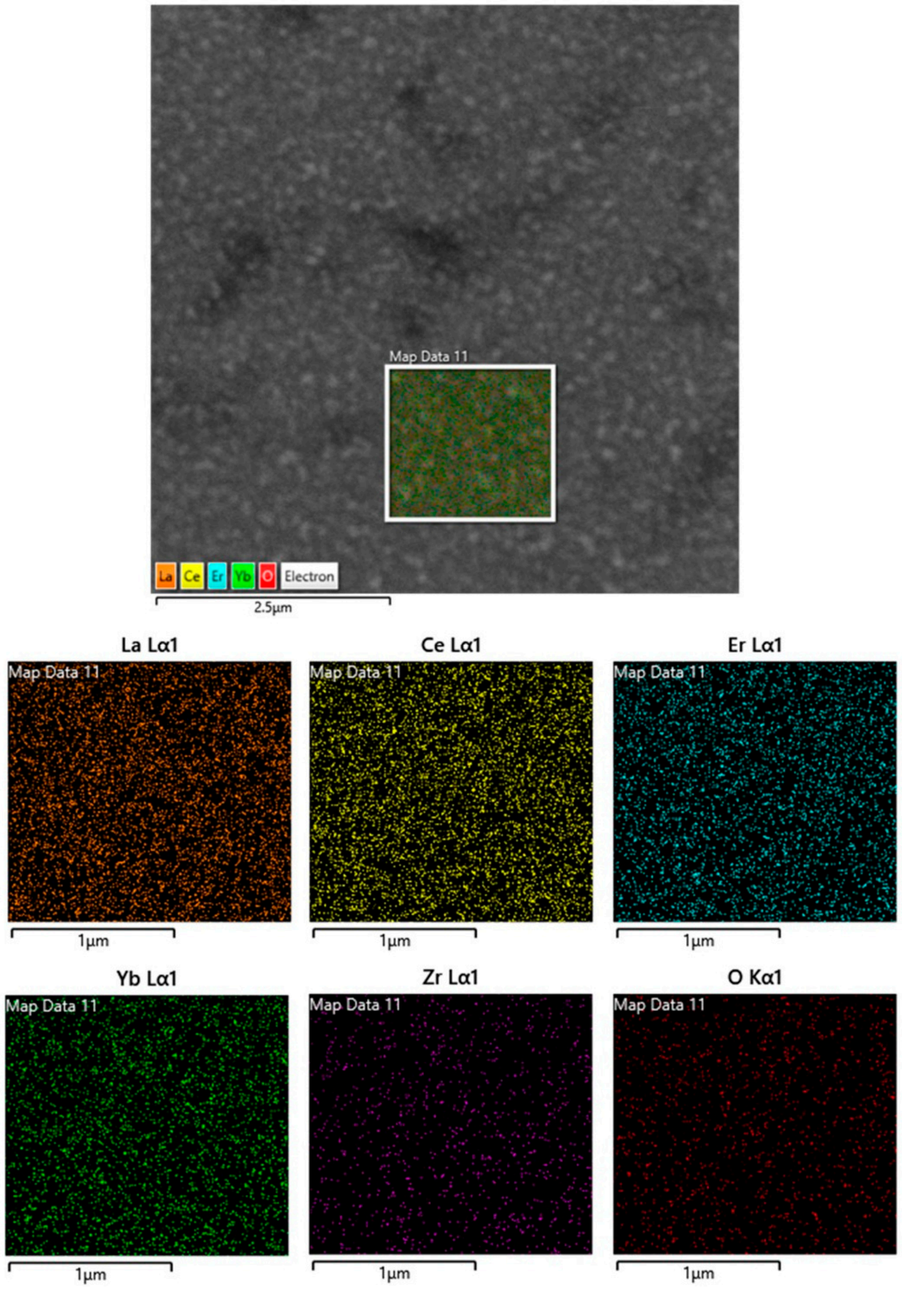

Figure S1. EDS maps of the exemplary CZYbEN-CP-s1300
